# An Open-Label Study of the Impact of Hepatic Impairment on the Pharmacokinetics and Safety of Single Oral and Intravenous Doses of Omadacycline

**DOI:** 10.1128/AAC.01650-20

**Published:** 2020-10-20

**Authors:** Steven J. Kovacs, Lillian Ting, Jens Praestgaard, Gangadhar Sunkara, Haiying Sun, Daniel S. Stein, S. Ken Tanaka, Stephen Villano

**Affiliations:** a Novartis Institute for BioMedical Research, Novartis Pharmaceuticals, East Hanover, New Jersey, USA; b Paratek Pharmaceuticals, Inc., King of Prussia, Pennsylvania, USA

**Keywords:** aminomethylcycline, omadacycline, hepatic impairment, pharmacokinetics

## Abstract

Omadacycline is a once-daily oral or intravenous (i.v.) aminomethylcycline antibiotic approved in the United States for the treatment of community-acquired bacterial pneumonia (CABP) and acute bacterial skin and skin structure infections (ABSSSI) in adults. Omadacycline pharmacokinetics were characterized in 18 patients with hepatic impairment and 12 matched healthy subjects. Patients with hepatic impairment received i.v. omadacycline at 100 mg (mild hepatic impairment) or 50 mg (moderate and severe hepatic impairment) and oral omadacycline at 300 mg (mild hepatic impairment) or 150 mg (moderate hepatic impairment); oral omadacycline was not evaluated in those with severe hepatic impairment. Safety monitoring included the collection of adverse events (AEs), performance of laboratory tests, determination of vital signs, and performance of electrocardiograms. Omadacycline exposures were similar in patients with hepatic impairment and healthy subjects following i.v. or oral administration, with the geometric mean ratios for the area under the concentration-time curve and the maximum drug concentration ranging from 0.79 to 1.42. Omadacycline was safe and well tolerated. Overall, 13/30 (43.3%) participants experienced an AE; those occurring in more than 1 participant included headache (13.3%), nausea (6.7%), infusion-site pain (6.7%), contusion (6.7%), and dizziness (6.7%), with no differences based on the degree of hepatic impairment or the route of administration. Asymptomatic increases in heart rate were observed; none was considered an AE. These findings suggest that no omadacycline dose adjustment is warranted in patients with hepatic impairment.

## Abstract (precis)

Omadacycline is a once-daily oral or intravenous (i.v.) aminomethylcycline antibiotic approved in the United States for the treatment of community-acquired bacterial pneumonia (CABP) and acute bacterial skin and skin structure infections (ABSSSI) in adults. Omadacycline pharmacokinetics were characterized in 18 patients with hepatic impairment and 12 matched healthy subjects. Patients with hepatic impairment received i.v. omadacycline at 100 mg (mild hepatic impairment) or 50 mg (moderate and severe hepatic impairment) and oral omadacycline at 300 mg (mild hepatic impairment) or 150 mg (moderate hepatic impairment); oral omadacycline was not evaluated in those with severe hepatic impairment.

## INTRODUCTION

Contemporary antibiotics often require dose adjustment for patients with some degree of hepatic impairment in order to maintain exposure largely within the range expected for the same dose given to patients who do not have hepatic impairment. Tigecycline, a glycylcycline tetracycline derivative, requires a dosage reduction by 50% in patients with severe hepatic impairment (1). Intravenous (i.v.) lefamulin, a pleuromutilin, requires a dosage reduction in patients with severe hepatic impairment; oral lefamulin has not been studied and is not recommended for patients with moderate or severe hepatic impairment (2). Among other antibiotic classes, oritavancin (a lipoglycopeptide) has not been evaluated in those with severe impairment (3); linezolid (an oxazolidinone) may require dosage adjustment in those with severe impairment (4); and clarithromycin (a macrolide), tedizolid (an oxazolidinone), and delafloxacin (a fluoroquinolone) require no dosage adjustment in patients with hepatic impairment (5). For drugs with hepatic elimination, increased systemic exposure from reduced hepatic clearance may lead to safety risks; therefore, clinical investigation of the effect of various degrees of hepatic impairment on pharmacokinetics (PK) is required to determine the need for dose adjustment (6).

Omadacycline is a once-daily oral or i.v. aminomethylcycline antibiotic that is approved in the United States for the treatment of adults with community-acquired bacterial pneumonia (CABP) and acute bacterial skin and skin structure infections (ABSSSI) (7). Omadacycline has activity against Gram-positive and Gram-negative aerobic bacteria, anaerobes, and atypical organisms (8, 9). In phase I studies of healthy adults, a 300-mg oral dose of omadacycline produced peak plasma concentrations exceeding 500 ng/ml and had an elimination half-life (*t*_1/2_) of approximately 17 h, thus supporting once-daily dosing (10). Oral bioavailability is 34.5% (10). Protein binding is approximately 21% in human serum (11). In phase III studies, omadacycline has shown efficacy as a once-daily oral and i.v. treatment for CABP and ABSSSI (12–14). Nonclinical data revealed that omadacycline is cleared hepatically (i.e., by biliary excretion) and renally (15). Clinical investigations indicate that no adjustment of the omadacycline dose is needed in patients with impaired renal function (16). The purpose of this study was to determine whether dose adjustment may be necessary for omadacycline in patients with hepatic impairment.

## RESULTS

Eighteen patients with mild (group 1), moderate (group 2), or severe (group 3) hepatic impairment and 12 healthy subjects were enrolled. Hepatic impairment was classified according to the Child-Turcotte-Pugh scoring method (17). Healthy subjects were matched to patients in either group 1 or group 2 (Table 1). All 30 participants were included in the safety analysis, and 29 (96.7%) completed the study; 1 patient in the mild impairment group discontinued due to an adverse event (AE) of rash after the i.v. dose in period 1 and did not receive the oral dose in period 2. Twenty-eight participants were included in the PK analysis set; 1 patient each in the mild and severe impairment groups was excluded because PK results were not reliable due to bioanalytical interference. A total of 23 participants received both the single i.v. dose and the single oral dose of omadacycline in the separate study periods, as planned. Per protocol, the 6 patients with severe hepatic impairment received a single i.v. dose of omadacycline only. At baseline, patients with hepatic impairment and healthy subjects were comparable for demographic characteristics (Table 2). All enrolled participants were white, the overall age (mean ± standard deviation) was 54.7 ± 5.4 years, and 26 (86.7%) participants were male.

**TABLE 1:** Treatment groups and assigned omadacycline doses^*d*^

Treatment group	Period 1, single i.v. dose (mg)	Period 2, single oral dose (mg)
Group 1, mild hepatic impairment (CTP class A^*a*^)	100	300
Group 2, moderate hepatic impairment (CTP class B^*b*^)	50	150
Group 3, severe hepatic impairment (CTP class C^*c*^)	50	
Group 4, healthy subject matched to group 1	100	300
Group 5, healthy subject matched to group 2	50	150

a Class A corresponds to a Child-Turcotte-Pugh score of 5 to 6.

b Class B corresponds to a Child-Turcotte-Pugh score of 7 to 9.

c Class C corresponds to a Child-Turcotte-Pugh score of 10 to 15.

d Each group had six subjects. One subject in group 1 (Child-Turcotte-Pugh class A) discontinued due to an adverse event of a rash after the i.v. dose in period 1 and did not receive the oral dose in period 2. CTP, Child-Turcotte-Pugh; i.v., intravenous.

**TABLE 2:** Baseline demographic characteristics

Characteristic	Value for the following subjects:			
	Hepatic impairment			Healthy (*n *= 12)
	Mild (*n *= 6)	Moderate (*n *= 6)	Severe (*n *= 6)	
Age (yr)				
Mean ± SD	54.0 ± 5.3	57.0 ± 6.2	56.2 ± 3.7	53.1 ± 5.8
Range	47–60	47–64	51–62	46–64
No. (%) of male subjects	5 (83.3)	6 (100)	4 (66.7)	11 (91.7)
Ht (cm)				
Mean ± SD	171.3 ± 3.8	174.7 ± 7.8	169.8 ± 11.1	174.0 ± 5.7
Range	165–175	166–188	152–182	166–184
Wt (kg)				
Mean ± SD	81.7 ± 13.9	80.6 ± 7.6	82.6 ± 21.6	80.5 ± 9.8
Range	63–102	71–93	63–118	64–94
BMI^*a*^ (kg/m^2^)				
Mean ± SD	27.9 ± 4.8	26.4 ± 1.2	28.4 ± 5.6	26.6 ± 2.9
Range	20.6–35.1	24.5–27.7	22.7–35.6	23.0–32.5
No. (%) of subjects of the following race/ethnicity				
Hispanic/Latino	2 (33.3)	4 (66.7)	3 (50.0)	7 (58.3)
White	6 (100)	6 (100)	6 (100)	12 (100)

a BMI, body mass index.

### Pharmacokinetics.

A comparison of plasma PK parameters for each group showed the expected dose-related differences in exposure for both the i.v. and oral routes of administration (Table 3). Generally, intersubject variability in exposure was higher following oral dosing than following the i.v. infusion. The median time to reach the peak concentration following drug administration (*T*_max_) ranged from 0.25 to 0.5 h with i.v. doses and from 1.5 to 2.0 h with oral doses. Similar clearance was observed across the groups: the mean total body clearance following i.v. administration (CL) ranged from 9.1 to 14.2 liters/h, and the mean apparent total body clearance following oral administration (CL/*F*) ranged from 48.4 to 72.1 liters/h. The terminal elimination half-life (*t*_1/2_) was relatively consistent and ranged from 8 to 16 h across the groups, with no apparent trend relative to the degree of hepatic impairment.

**TABLE 3:** Plasma pharmacokinetic parameters for omadacycline by treatment group^*a*^

Parameter	Value (no. of subjects) for:								
	Patients with hepatic impairment					Healthy matched controls			
	Mild (group 1)		Moderate (group 2)		Severe (group 3), 50 mg i.v.	Matched to mild (group 4)		Matched to moderate (group 5)	
	100 mg i.v.	300 mg oral	50 mg i.v.	150 mg oral		100 mg i.v.	300 mg oral	50 mg i.v.	150 mg oral
AUC_last_ (ng · h/ml)	9,734 ± 1,943 (5)	5,839 ± 2,765 (4)	3,542 ± 397 (6)	3,213 ± 828 (6)	4,484 ± 531 (5)	10,851 ± 2,595 (6)	6,533 ± 1,665 (6)	4,199 ± 721 (6)	3,162 ± 1,033 (6)
AUC_inf_ (ng · h/ml)	9,734 ± 1,943 (5)	5,839 ± 2,765 (4)	3,542 ± 397 (6)	3,213 ± 828 (6)	4,484 ± 531 (5)	10,851 ± 2,595 (6)	6,533 ± 1,665 (6)	4,199 ± 721 (6)	3,162 ± 1,033 (6)
*C*_max_ (ng/ml)	9,734 ± 1,943 (5)	5,839 ± 2,765 (4)	3,542 ± 397 (6)	3,213 ± 828 (6)	4,484 ± 531 (5)	10,851 ± 2,595 (6)	6,533 ± 1,665 (6)	4,199 ± 721 (6)	3,162 ± 1,033 (6)
*T*_max_ (h)^*b*^	9,734–1,943 (5)	5,839–2,765 (4)	3,542–397 (6)	3,213–828 (6)	4,484–531 (5)	10,851–2,595 (6)	6,533–1,665 (6)	4,199–721 (6)	3,162–1,033 (6)
*t*_1/2_ (h)	9,734 ± 1,943 (5)	5,839 ± 2,765 (4)	3,542 ± 397 (6)	3,213 ± 828 (6)	4,484 ± 531 (5)	10,851 ± 2,595 (6)	6,533 ± 1,665 (6)	4,199 ± 721 (6)	3,162 ± 1,033 (6)
CL or CL/*F* (liters/h)^*c*^	9,734 ± 1,943 (5)	5,839 ± 2,765 (4)	3,542 ± 397 (6)	3,213 ± 828 (6)	4,484 ± 531 (5)	10,851 ± 2,595 (6)	6,533 ± 1,665 (6)	4,199 ± 721 (6)	3,162 ± 1,033 (6)
*V_z_* or *V_z_*/*F* (liters)^*c*^	9,734 ± 1,943 (5)	5,839 ± 2,765 (4)	3,542 ± 397 (6)	3,213 ± 828 (6)	4,484 ± 531 (5)	10,851 ± 2,595 (6)	6,533 ± 1,665 (6)	4,199 ± 721 (6)	3,162 ± 1,033 (6)

a Data are for 28 subjects. Values are the mean ± standard deviation unless otherwise stated. AUC_inf_, area under the concentration-time curve from time zero to infinity; AUC_last_, area under the concentration-time curve from time zero to the last quantifiable concentration point; CL, mean total body clearance following intravenous administration; CL/*F*, apparent total body clearance following oral administration; *C*_max_, maximum drug concentration; i.v., intravenous; *t*_1/2_, terminal elimination half-life; *T*_max_, time to reach peak concentration following drug administration; *V_z_*, apparent volume of distribution (beta method) following i.v. administration; *V_z_*/*F*, apparent volume of distribution (beta method) following oral administration.

b *T*_max_ is reported as the median (range).

c CL and *V_z_* for i.v. infusion; CL/*F* and *V_z_*/*F* for oral administration.

Overall, the geometric mean ratios for the comparison of patients with hepatic impairment to the matched healthy control subjects showed that omadacycline exposure was similar regardless of the severity of hepatic impairment (Table 4). Following single oral and i.v. doses of omadacycline, the area under the concentration-time curve (AUC) from time zero to infinity (AUC_inf_), the area under the concentration-time curve from time zero to the last quantifiable concentration point (AUC_last_), and the maximum drug concentration (*C*_max_) in patients with hepatic impairment were comparable to those in healthy subjects (Table 4). While a somewhat higher *C*_max_ was observed following i.v. dosing of 100 mg in patients with mild hepatic impairment than in the matched controls, this trend was not observed in patients with moderate or severe hepatic impairment.

**TABLE 4:** Geometric mean ratio for primary pharmacokinetic parameters after i.v. or oral omadacycline in patients with hepatic impairment versus healthy subjects^*d*^

Parameter	Geometric mean ratio (90% confidence interval) for:				
	Group 1 (mild hepatic impairment)^*a*^		Group 2 (moderate hepatic impairment)^*b*^		Group 3 (severe hepatic impairment)^*c*^
	100 mg i.v.	300 mg oral	50 mg i.v.	150 mg oral	50 mg i.v.
AUC_last_ (ng · h/ml)	0.90 (0.73, 1.11)	0.79 (0.50, 1.24)	0.85 (0.75, 0.97)	1.02 (0.75, 1.40)	1.08 (0.91, 1.27)
AUC_inf_ (ng · h/ml)	0.86 (0.69, 1.07)	0.79 (0.50, 1.24)	0.88 (0.78, 0.99)	1.02 (0.75, 1.40)	1.08 (0.91, 1.27)
*C*_max_ (ng/ml)	1.42 (1.10, 1.84)	0.96 (0.64, 1.42)	1.02 (0.84, 1.25)	1.24 (0.94, 1.65)	1.08 (0.89, 1.31)

a Group 1, mild hepatic impairment versus matched healthy subjects.

b Group 2, moderate hepatic impairment versus matched healthy subjects.

c Group 3, severe hepatic impairment versus healthy subjects matched to group 2 receiving omadacycline at 50 mg i.v.

d AUC_inf_, area under the concentration-time curve from time zero to infinity; AUC_last_, area under the concentration-time curve from time zero to the last quantifiable concentration point; *C*_max_, maximum drug concentration in plasma; i.v., intravenous.

**FIG 1:**
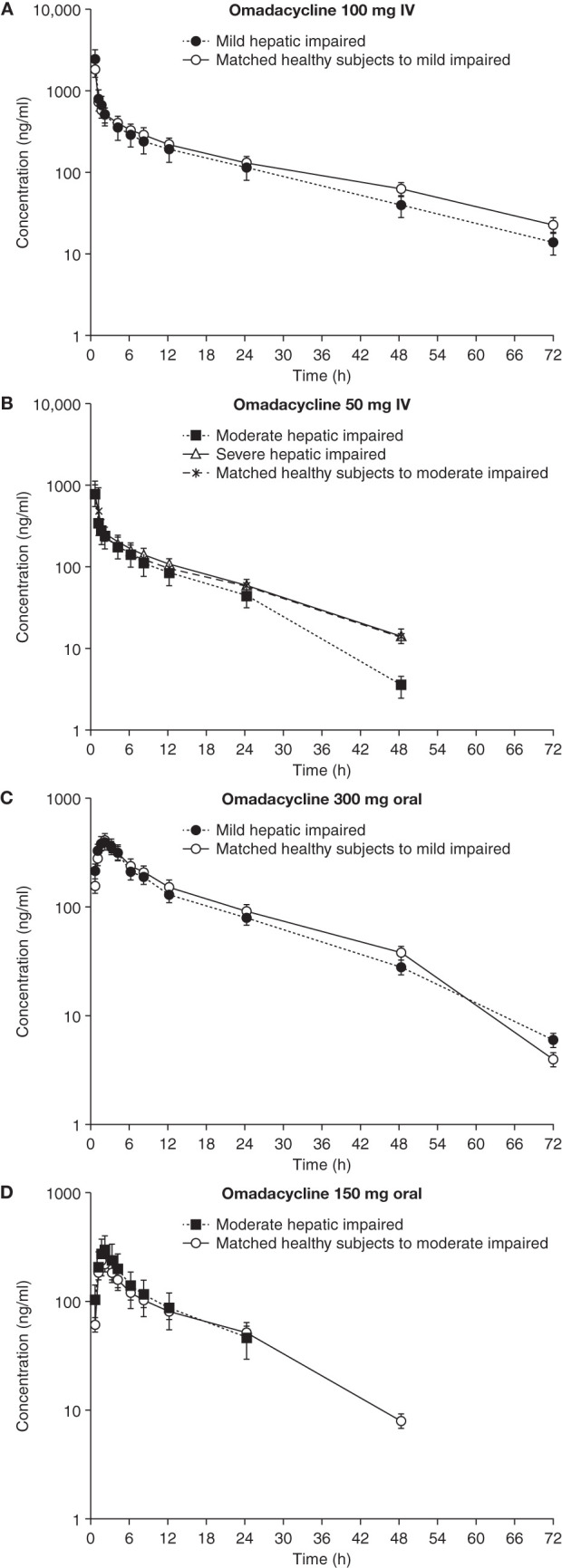
Plasma concentration-time profiles for omadacycline following i.v. (A and B) and oral (C and D) administration in patients with hepatic impairment versus healthy subjects. Data are shown as the mean ± standard deviation. IV, intravenous.

**FIG 2:**
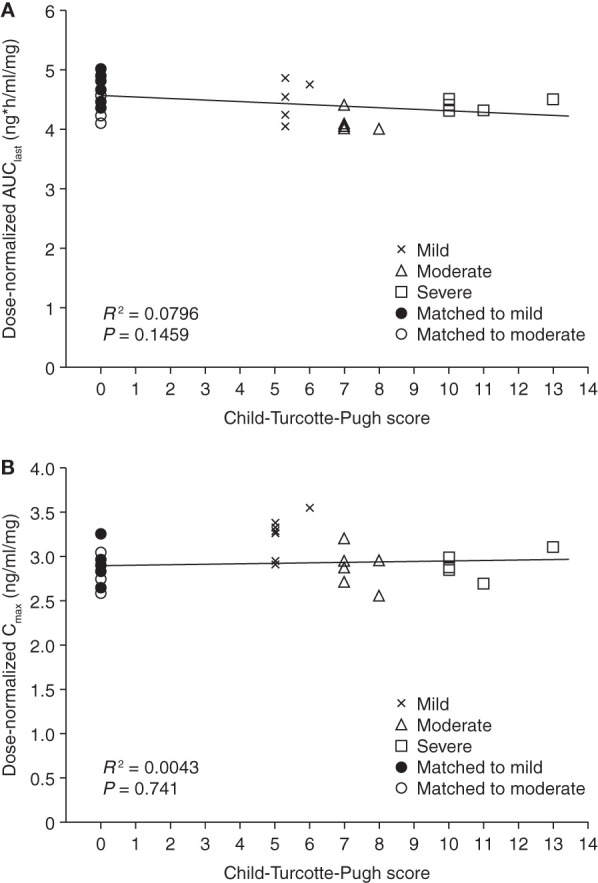
Dose-normalized AUC_last_ (A) and *C*_max_ (B) versus Child-Turcotte-Pugh score following an intravenous infusion of omadacycline (healthy subjects were assigned a score of 0). AUC_last_, area under the concentration-time curve from time zero to the last quantifiable concentration point; *C*_max_, maximum drug concentration.

The mean plasma concentration-time profiles for i.v. omadacycline at 50 and 100 mg and oral omadacycline at 150 and 300 mg were comparable for patients with mild, moderate, or severe hepatic impairment and the matched healthy subjects (Fig. 1). The dose-normalized AUC_last_ and *C*_max_ following i.v. omadacycline demonstrated no relationship (the *R*^2^ correlation values for regression analysis were approximately 0) between exposure to omadacycline and the severity of hepatic impairment (Fig. 2).

### Safety/tolerability.

Overall, 13 (43.3%) participants experienced at least 1 AE. AE rates were similar between patients with hepatic impairment and healthy subjects. The most commonly reported AEs were headache (13.3%), nausea (6.7%), infusion-site pain (6.7%), contusion (6.7%), and dizziness (6.7%). All other AEs were reported in only 1 participant. One patient, a 47-year-old male with moderate hepatic impairment, experienced serious AEs of alcohol intoxication with angina pectoris, hypocalcemia, hypotension, and rhabdomyolysis; these occurred 8 days after receiving oral omadacycline at 150 mg. He had previously received i.v. omadacycline at 50 mg, which he tolerated well. This patient had a history of alcoholic cirrhosis, hepatitis C, hypertension, and diabetes mellitus, among other conditions. The events resolved after supportive care in the hospital; none of these events was considered related to omadacycline. One patient with mild hepatic impairment discontinued the study due to a mild facial rash, which occurred several hours after receiving i.v. omadacycline at 100 mg. This event was considered related to omadacycline; the rash was treated with diphenhydramine and resolved after approximately 4 days. Other than the laboratory findings in the patient with the serious AEs described above, there were no clinically relevant changes in serum chemistry, hematology, or physical examination findings during the study.

Participants in all treatment groups experienced a mean maximum increase in heart rate (from predose to any postdose measurement) that ranged from 3 to 19 beats per minute. Mean maximum increases in systolic and diastolic blood pressure ranged from 8 to 13 mm Hg and 3 to 10 mm Hg, respectively. Increases in heart rate and blood pressure were transient; they typically occurred within 6 to 12 h after dosing, returned to normal levels by 24 h after the dose, and were not reported as AEs. Electrocardiogram (ECG) data reflected the heart rate changes but showed no other notable findings. There were no apparent differences in these effects between the patients with hepatic impairment and the healthy subjects; heart rate changes tended to occur earlier following i.v. administration than following oral administration.

## DISCUSSION

Omadacycline is incompletely absorbed from the gastrointestinal tract, resulting in an oral bioavailability of about 35%. Systemically available omadacycline undergoes negligible biotransformation and is eliminated largely unchanged in feces and urine (15). Following oral administration, excretion of unabsorbed drug in feces is the predominant route of elimination of omadacycline, with approximately 14% of the total dose and 40% of the absorbed dose being excreted in urine (15, 16). The results of this study suggest that hepatic impairment does not have a clinically relevant impact on the PK of omadacycline following oral or i.v. administration. The dose-normalized AUC and *C*_max_ across all study groups after i.v. administration showed no association between exposure to omadacycline and the Child-Turcotte-Pugh score. Different degrees of hepatic impairment had no clinically relevant effect on the systemic clearance of i.v. omadacycline.

Although it was unexpected that hepatic impairment had only a small effect on the clearance of omadacycline, such an effect is not unprecedented. For example, in an open-label, parallel-group, PK study, the metabolism of oseltamivir was not compromised in subjects with moderate hepatic impairment (18). In addition, in 2 open-label, single-dose studies investigating the effect of hepatic or renal impairment on the PK of aripiprazole, no meaningful differences in aripiprazole PK were found between groups of subjects with normal hepatic or renal function and those with hepatic or renal impairment (19). We can only speculate as to why the PK of omadacycline were not substantially affected in hepatically impaired subjects across the Child-Turcotte-Pugh score classes. It is possible that a lower hepatic clearance of omadacycline in these subjects was compensated for by an increase in renal clearance. However, urine samples were not taken in the study to determine renal clearance. Another possible explanation is that the levels of hepatic impairment in these individuals did not affect the biliary transport functions to such an extent to affect the overall clearance of omadacycline. At this time, it is not known how omadacycline is taken up by hepatocytes or secreted across the biliary canalicular membranes. Omadacycline has been shown *in vitro* not to be a substrate of several known biliary and renal transporters, such as OATP1B1, OATP1B3, MRP2, and BCRP (20).

Single doses of omadacycline were safe and well tolerated, with no apparent differences between healthy subjects and patients with hepatic impairment, although the sample sizes were small. The only AE that occurred in more than 2 study participants was headache (which occurred in 4 participants [13%]). Modest, asymptomatic increases in heart rate were observed for several hours after dose administration, but no increase was considered to be an AE or associated with ECG abnormalities. Asymptomatic increases in heart rate have also been observed in other phase I clinical studies of omadacycline (10, 21), which is thought to be due to a vagolytic effect (omadacycline does not bind to adrenergic receptors and has no direct effect on the sinoatrial node) (21). To date, this finding has not been associated with cardiac arrhythmia or other clinically significant cardiovascular toxicity in clinical studies (21).

A possible limitation of this study is that oral omadacycline was not administered to patients with severe hepatic impairment. If a drug is to be administered by more than 1 route, only the route that provides the most information about the impact of hepatic impairment on the drug’s elimination needs to be studied (6). Omadacycline is neither extensively metabolized nor protein bound, and the results of this study revealed no relevant impact of any degree of hepatic impairment on omadacycline elimination following i.v. administration. The results of this study, however, cannot address the potential impact, if any, of severe hepatic impairment on absorption following oral administration because it was not specifically investigated.

Older antibiotics in the tetracycline class (e.g., tetracycline, oxytetracycline, minocycline, and doxycycline) have generally not been studied or specifically labeled for use in patients with hepatic impairment (22–24). Data on the PK of tigecycline in patients with hepatic impairment are available (1). No clinically relevant difference in PK was observed between healthy subjects and patients with mild hepatic impairment, but the total exposure AUC increased by 50% and 105% in those with moderate and severe hepatic impairment, respectively, necessitating a recommendation for a tigecycline dose reduction in those with severe impairment (1).

With respect to nontetracycline antibiotics, reduced doses of i.v. lefamulin (a pleuromutilin antibacterial) are required in patients with severe hepatic impairment (2). Lefamulin tablets have not been studied in patients with moderate or severe hepatic impairment and are not recommended (2). The antimycobacterial agent rifampicin is contraindicated in patients with hepatic dysfunction (25).

The potential for hepatotoxicity is well documented with some antibiotics, including tetracyclines, clavulanic acid, macrolides, sulfonamides, and linezolid; however, hepatotoxicity is rare for most other classes of antibiotics (26). The risk for hepatotoxicity is an additional consideration that can be independent from dose selection to achieve similar systemic exposures. An integrated analysis of safety for 3 phase III clinical trials of omadacycline showed that the rate of occurrence of treatment-associated liver-related AEs (5.4%) was comparable to that seen with linezolid (4.9%) and moxifloxacin (7.2%) (27). Moreover, the observed changes from baseline for the transaminases associated with exposure to omadacycline were not considered clinically significant. The absence of a signal for possibly different safety across the different hepatic impairment classes in this small study is consistent with the findings of the integrated safety analyses and the prior clinical experience with omadacycline. The results of this study suggest that systemic omadacycline exposures following i.v. administration of 100 mg or oral administration of 300 mg—the bioequivalent therapeutic doses for treatment of CABP and ABSSSI—can be expected to be similar in those with and those without liver disease.

The availability of once-daily oral and i.v. dosing and the lack of a need for dosage adjustment with omadacycline in populations with liver disease offer simplicity and possible advantages for its use in the treatment of common bacterial infectious diseases.

## MATERIALS AND METHODS

The study was conducted at 2 centers in the United States (the Orlando Clinical Research Center and the University of Miami) according to the ethical principles of the Declaration of Helsinki and good clinical practice. The study protocol, consent form, and all amendments were reviewed and approved by an independent ethics committee or institutional review board for each center (Independent Investigational Review Board, Inc., and the University of Miami Human Subjects Research Office). Written informed consent was obtained from each study participant prior to enrollment.

### Study design.

This was an open-label, fixed-sequence study in adult patients with hepatic impairment and healthy adult subjects. The degree of hepatic impairment was classified according to the Child-Turcotte-Pugh scoring method (17). Two separate groups of healthy subjects were matched to patients with mild or moderate hepatic impairment by age (±10 years), sex, weight (±10 kg), and smoking status (Table 1).

The study consisted of a screening period that did not exceed 28 days, a baseline period, and 2 treatment periods. During period 1, patients with mild hepatic impairment (group 1) received a single i.v. dose of omadacycline at 100 mg, and those with moderate and severe hepatic impairment (groups 2 and 3) received a single i.v. dose of omadacycline at 50 mg; omadacycline was administered as a 30-min i.v. infusion. A washout period of at least 7 days separated periods 1 and 2. During period 2, groups 1 and 2 received a single oral dose of omadacycline at 300 mg or 150 mg, respectively; they were required to fast for 10 h before and 4 h after the dose. In both periods, healthy subjects matched to groups 1 and 2 received the same doses as the subjects in their respective hepatic impairment groups (Table 1). An end-of-study evaluation was performed approximately 1 week after dosing in period 2. Lower doses of omadacycline were administered to patients in groups 2 and 3 as a precaution, given that the biliary excretion of unchanged drug is the major component of elimination and that the clearance of tigecycline (a compound that is also derived from tetracyclines) is reduced and requires a dose reduction in patients with severe hepatic impairment (1).

### Subject selection.

Healthy control subjects were eligible if they were aged 18 to 70 years, weighed at least 50 kg (body mass index, 18 to 36 kg/m^2^), had normal vital signs with no orthostatic changes, and were in good health generally. Patients with hepatic impairment had to meet the same criteria for age, weight, and vital signs; they also had to have a Child-Turcotte-Pugh score of at least 5 and to have been in a stable condition for at least 3 months prior to enrollment. In all groups, the following exclusion criteria were applied: tobacco use (>10 cigarettes per day), recent blood donation or a hemoglobin concentration of <10 g/dl, a history of hypersensitivity to omadacycline or similar drugs (tetracyclines), a history of malignancy, and a history of any medical condition that could interfere with the conduct of the study. Pregnant or lactating women were excluded, and women of childbearing potential had to use an acceptable form of contraception. Healthy control subjects were also excluded for the following reasons: clinically significant ECG abnormalities; a history or presence of impaired renal function; and the use of other prescription medications, herbal supplements, over-the-counter drugs, or investigational drugs. Patients with hepatic impairment were also excluded for a calculated creatinine clearance of <50 ml/min or the use of investigational drugs; these patients could receive their routine prescription or over-the-counter medications, but drugs such as antacids, calcium-containing supplements, sucralfate, lactulose, and binding resins could not be taken for at least 12 h before and after the administration of omadacycline.

### Study assessments.

Peripheral blood samples (4 ml each), obtained by direct venipuncture or an indwelling cannula inserted in a forearm vein, were collected prior to dose administration and at 0.25, 0.5, 1, 1.5, 2, 3, 4, 6, 8, 12, 24, 48, 72, and 96 h after dose administration in tubes containing sodium heparin. The samples were centrifuged for 10 min within 30 min of collection. The resulting plasma was transferred to polypropylene screw-cap tubes and placed on dry ice. The tubes were kept frozen at −70°C or colder until analysis. The plasma concentrations of omadacycline were measured using a validated liquid chromatography-mass spectrometry/mass spectrometry assay having a lower limit of quantitation of approximately 20 ng/ml. Calibration standard responses were linear over the range of 20 to 2,000 ng/ml. In plasma, the interday assay accuracy, expressed as percent relative error for quality control (QC) concentrations (20, 60, 758, and 1,520 ng/ml), ranged from −5.3% to 2.0% bias in QC samples. Assay precision, expressed as the interday percent coefficients of variation of the mean estimated concentrations of the QC samples, ranged from 3.1% to 4.5%. The extraction efficiency ranged from 87.0% to 93.5% across the QC samples. The overall mean matrix effect at 60, 758, and 1,520 ng/ml was 1.8%.

At screening and baseline (the day prior to dosing), the study participants underwent a physical examination; determination of vital signs (temperature, blood pressure, and heart rate), height (at screening only), and body weight; a serum or urine pregnancy test; and tests for alcohol and drugs. Blood pressure and heart rate were measured at screening, baseline, and predose, as well as at multiple time points on the day of dosing and at the end-of-study visit. A urinalysis was performed at screening, as were hepatitis virus and HIV tests. A 12-lead ECG was obtained at screening and baseline; at 2, 12, and 24 h after dosing; and at the end-of-study visit. Serum chemistry and hematology testing were carried out at screening and baseline, at 24 and 48 h after dosing, and at the end-of-study visit. Safety assessments consisted of collecting all AEs and serious AEs, noting their severity and relationship to the study drug.

### Pharmacokinetic analysis.

The plasma concentrations of omadacycline are reported in nanograms per milliliter. Except for the predose samples, concentrations below the limit of quantitation were excluded from the calculation of the PK parameters. Plasma concentration-time data were analyzed using standard noncompartmental methods incorporating SAS programs validated with WinNonlin software (Certara USA, Inc., Princeton, NJ). PK parameters included AUC_last_ and AUC_inf_, each of which was calculated by the linear trapezoidal linear interpolation method; *C*_max_; CL; apparent total body clearance following oral administration (CL/*F*); apparent volume of distribution (beta method) following i.v. administration (*V_z_*); apparent volume of distribution (beta method) following oral administration (*V_z_*/*F*); *T*_max_; and *t*_1/2_, calculated from the PK program that included a minimum of 3 terminal time points.

### Statistical analysis.

The primary PK analysis variables were AUC_last_, AUC_inf_, and *C*_max_. Secondary variables were *T*_max_, CL or CL/*F*, *V_z_* or *V_z_*/*F*, and *t*_1/2_. The numerical Child-Turcotte-Pugh score (17) was used to correlate liver function with the dose-normalized PK exposure.

AUC and *C*_max_ were analyzed for patients with mild and moderate hepatic impairment and their matched healthy controls, with a fixed effect of hepatic function (mild impairment or normal) and a random effect for matching block or impaired subject/healthy subject pair. The 90% confidence intervals were calculated for the ratio of PK parameters for impaired versus healthy subjects. All analyses were done on the logarithmic scale and back-transformed for reporting. Analysis was performed separately for the i.v. and oral dose parameters. Dose-normalized AUC values after a single i.v. infusion were pooled across all study groups, and the relationship to the Child-Turcotte-Pugh score was investigated by exploratory regression. Various linear and polynomial regressions were explored to find a well-fitting statistical model for this relationship.

### Data availability.

Paratek Pharmaceuticals, Inc., has a commitment to ensure that access to clinical trial data is available to regulators, researchers, and trial participants, when permitted, feasible, and appropriate. Requests for deidentified patient-level data may be submitted to medinfo@paratekpharma.com for review.
